# Multimodal Sensor-Input Architecture with Deep Learning for Audio-Visual Speech Recognition in Wild

**DOI:** 10.3390/s23041834

**Published:** 2023-02-07

**Authors:** Yibo He, Kah Phooi Seng, Li Minn Ang

**Affiliations:** 1 School of AI and Advanced Computing, Xian Jiaotong Liverpool University, Suzhou 215123, China; 2 School of Computer Science, Queensland University of Technology, Brisbane, QLD 4000, Australia; 3 School of Science, Technology and Engineering, University of Sunshine Coast, Sippy Downs, QLD 4502, Australia

**Keywords:** multimodal sensing, audio-visual speech recognition, deep learning

## Abstract

This paper investigates multimodal sensor architectures with deep learning for audio-visual speech recognition, focusing on in-the-wild scenarios. The term “in the wild” is used to describe AVSR for unconstrained natural-language audio streams and video-stream modalities. Audio-visual speech recognition (AVSR) is a speech-recognition task that leverages both an audio input of a human voice and an aligned visual input of lip motions. However, since in-the-wild scenarios can include more noise, AVSR’s performance is affected. Here, we propose new improvements for AVSR models by incorporating data-augmentation techniques to generate more data samples for building the classification models. For the data-augmentation techniques, we utilized a combination of conventional approaches (e.g., flips and rotations), as well as newer approaches, such as generative adversarial networks (GANs). To validate the approaches, we used augmented data from well-known datasets (LRS2—Lip Reading Sentences 2 and LRS3) in the training process and testing was performed using the original data. The study and experimental results indicated that the proposed AVSR model and framework, combined with the augmentation approach, enhanced the performance of the AVSR framework in the wild for noisy datasets. Furthermore, in this study, we discuss the domains of automatic speech recognition (ASR) architectures and audio-visual speech recognition (AVSR) architectures and give a concise summary of the AVSR models that have been proposed.

## 1. Introduction

Automatic speech recognition (ASR) by machine has been a field of research for more than 60 years. It is robust against the full range of real-world noise and other acoustic distorting conditions. However, reliably recognizing spoken words in realistic acoustic environments is still a challenge. Audio-visual speech recognition (AVSR) is the task of transcribing text from audio and visual streams, which has recently attracted a lot of research attention due to its robustness against noise. Since the visual stream is not affected by the presence of noise, an audio-visual model can lead to improved performance over an audio-only model as the level of noise increases.

Previous works and studies in sensor-input architectures for automatic speech recognition (ASR) applications can be characterized by two approaches: (1) shallow-structured learning approaches for network training and classification; and (2) the utilization of singular modalities, such as speech signals, for training network architectures. Artificial neural network (ANN) architectures, such as the multilayer perceptron (MLP), radial basis function (RBF) networks and support vector machines (SVMs), are shallow-structured learning architectures which have been frequently used for ASR (Haton 1999 [1]; Phillips, Tosuner and Robertson, 1995 [2]) and other multimedia applications, such as face recognition (Lim et al., 2009 [3]).

Shallow-structured learning architectures are simple network structures that typically only have a single hidden layer for nonlinear feature transformations. Other examples of shallow-structured learning architectures that have been used for ASR are hidden Markov models (HMMs; Kinjo and Funaki, 2006 [4]), conditional random fields (CRFs; Zweig and Nguyen, 2009 [5]) and Gaussian mixture models (GMMs) (Fujimoto and Riki, 2004 [6]). In 2016, Deng [7] commented that “shallow architectures have been shown effective in solving many simple or well-constrained problems, but their limited modeling and representational power can cause difficulties when dealing with more complicated real-world applications involving natural signals such as human speech, natural sound and language, and natural image and visual scenes”.

The first set of advancements in ASR was achieved by using deep neural network (DNN) architectures and learning approaches. Compared to shallow-structured learning approaches, deep learning architectures have the characteristics of utilizing several (hundreds to thousands) layers of nonlinear computational stages that are organized in a hierarchical structure to enable the end-to-end learning of complex systems. These DNN architectures have been shown to overcome the local optimization issues that are typically encountered in shallow-structured learning-neural-network architectures. Deng [7] and Nassif et al. [8]) present surveys of deep-learning architectures for ASR applications. The second set of advancements in ASR was achieved by using multimodal sensor inputs for the architectures. Compared to single-modality ASR-sensor architectures and approaches, multimodal sensor architectures have the characteristics of utilizing multiple input modalities (e.g., speech, image, biosignals, etc.) to enable higher classification performance and robustness against noise.

When both the speech and image modalities are utilized for ASR tasks, the problem is often termed audio-visual speech recognition (AVSR). Dupont and Luettin [9] discuss a typical structure for an AVSR multimodal-sensor architecture. Their AVSR architecture consists of three components: (1) an acoustic or audio module to extract noise-robust features from the speech signal; (2) an image or visual module to extract lip-contour information and color information on the mouth area; and (3) a fusion module using HMMs for the combined modeling of the audio- and visual-feature streams. Note that the usage of audio and visual modalities is a broad research area and has been proposed for several applications, such as biometrics (Aleksic and Katsaggelos [10]), human-emotion recognition (Seng and Ang [11]) and event localization (Tian et al. [12]).

Although several advancements have been made in terms of the deep-learning structures and usage of multiple modalities for AVSR architectures, several challenges remain to be resolved for AVSR applications in the wild. The term “in the wild” is used to describe AVSR for unconstrained natural-language audio streams and video-stream modalities (Son Chung et al. [13]). Compared to datasets taken under fixed laboratory conditions, in the wild represents a different range of individuals, poses, expressions, lighting conditions and time frames in real environments. Some representative works using AVSR applications in the wild include those of Yu et al. [14]; Afouras et al., 2018 [15]). The next section will present further discussions on these and other related works for ASR and AVSR research and multimodal sensor architectures for speech recognition in the wild environment.

The aim of this paper is to contribute towards AVSR research from different perspectives. First, we discuss the domains of automatic speech recognition (ASR) and audio-visual speech recognition (AVSR) and give a concise summary of the AVSR models that have been proposed. Second, we propose new improvements for AVSR models by incorporating data-augmentation techniques to generate additional data samples for constructing classification models. For the data-augmentation techniques, we utilized a combination of conventional approaches (e.g., flips and rotations), as well as newer approaches, such as generative adversarial networks (GANs), to demonstrate the potential of these approaches. GANs are algorithmic architectures that use two neural networks, pitting one against the other in order to generate new, synthetic instances of data that can pass for real data. Shoumy et al. [16]) proposed the utilization of data-augmentation approaches for improving speech-emotion-classification tasks. In our work, we propose to incorporate data-augmentation techniques into AVSR models and frameworks for improved performance, targeting in-the-wild scenarios.

This paper is organized according to the following structure. Section 2 discusses some related works on multimodal-sensor architectures for audio-visual speech recognition for wild and not-wild environments. Section 3 describes the methodology, SR architecture by speech modality, SR architecture by facial modality and fusion-based AVSR approaches used for the investigation. Section 4 discusses the results. Section 5 presents some concluding remarks.

## 2. Related Works on Multimodal-Sensor Architectures for Speech Recognition in Wild Environments

This section provides some background information before we discuss our proposed approach for multimodal-sensor architectures for audiovisual speech recognition in the wild environment. Although speech recognition and visual recognition are mature research areas, comparatively few authors have proposed techniques to deal with ASR and AVSR tasks for in-the-wild scenarios. Table 1 shows a summary of some representative works for ASR, AVSR and other applications for in-the-wild scenarios. We provide examples for four categories or domains: (1) research works on ASR (audio-only, singular modality) in the wild; (2) research works on AVSR (audio-visual, multiple modalities) in the wild; (3) research works for emotion recognition in the wild; and (4) research works on other in-the-wild applications. The purpose of the work proposed in this paper is to contribute towards research for multiple-modality AVSR with a focus on in-the-wild environments.

### 2.1. Research Works for ASR in the Wild

Some examples of representative works for ASR in the wild are those of Stafylakis and Tzimiropoulos [17], Han et al. [18] and Ali et al. [19]. Stafylakis and Tzimiropoulos [17] proposed an approach for visual speech recognition for in-the-wild scenarios. Their approach used an end-to-end zero-shot learning architecture for visual-only keyword spotting (KWS) tasks and had three components: (1) a visual-feature extractor using spatiotemporal residual networks; (2) a grapheme-to-phoneme encoder–decoder model utilizing sequence-to-sequence neural networks; and (3) recurrent neural networks for visual-feature correlation. Their experimental results on the LRS2 dataset showed that their proposed architecture offers a promising level of performance for visual-only KWS tasks on the LRS2 dataset.

Han et al. [18] proposed an approach to telephony-speech recognition for in-the-wild tasks utilizing deep-learning techniques. Their work evaluated three deep-learning architectures: (1) time-delay neural network (TDNN); (2) bidirectional long short-term memory (BLSTM); and (3) convolutional neural network BLSTM (CNN–BLSTM)). Their experimental results utilized the Switchboard and CallHome datasets and showed that the CNN–BLSTM deep -earning architecture outperformed the other two models and demonstrated good performance for the in-the-wild and real-world telephony datasets.

Ali, Vogel and Renals [19] offer another approach to ASR research in the wild. Their work describes their attempt to apply ASR in the Arabic language. They term this the Arabic MGB-3 Challenge. The dataset used for this challenge contains a challenging mixture collected from YouTube videos from seven genres (comedy, cooking, family/kids, fashion, drama, sports and science), consisting of 16 h of videos. The authors received submissions from 13 teams for the challenge. Their work showed that the best submission using a combined approach of GANs and lexical information could achieve a performance accuracy of 80% across five classes.

### 2.2. Research Works for AVSR in the Wild

Some examples of representative works for AVSR in the wild include those of Yu et al. [14], Afouras et al. [15] and Son Chung et al. [13]). Yu et al. [14] proposed an approach to AVSR in the wild for overlapped speech with interfering speakers. Their approach used time-delay neural networks (TDNNs) with a lattice-free MMI (LF–MMI) discriminative criterion (termed as LF–MMI TDNN system). The authors proposed two architectures for AVSR: (1) a hybrid AVSR architecture and (2) an end-to-end AVSR architecture. Their experiments using the LRS2 dataset showed that the hybrid AVSR architecture gave a better performance than the end-to-end AVSR architecture. The authors showed that their proposed AVSR architecture can outperform an audio-only baseline ASR architecture by around 29% in terms of the WER (word-error rate).

Afouras et al. [15] proposed an approach for AVSR in the wild utilizing deep-learning techniques and transformer-based models. The authors proposed two transformer (TM) models and architectures for AVSR: (1) an encoder–decoder-attention-structure TM architecture and (2) a self-attention TM-stack architecture. The self-attention transformer stack architecture was termed TM–CTC and consisted of multiple stacks of self-attention and feedforward layers, which generated the posterior probabilities for connectionist temporal classification (CTC) loss. The experimental results on the LRS2-BBC dataset showed that the proposed TM AVSR architectures could give a reduced WER of 8.2% compared with an audio-only baseline AVSR architecture, which gave a WER of 10.1%.

Son Chung et al. [13]) offer another approach to AVSR research in the wild. This work describes the authors’ AVSR architecture, termed the watch, listen, attend and spell (WLAS) network model, which has the ability to transcribe speech into characters. Their WLAS architecture can be configured to utilize attention models for visual input (VO) only, audio input (AO) only and audio-visual (AV) input. Their experimental results on the Lip-Reading in the Wild (LRW) and GRID datasets showed that their proposed AVSR architecture gave a reduced WER of 23.8% and 3.0% for the LRW and GRID datasets, respectively, compared with other approaches.

### 2.3. Research Works on Emotion Recognition in the Wild

Some examples of representative works on emotion recognition in the wild include those by Li et al. [20], Lu et al. [21] and Chen et al. [22]). The authors in Li et al. [20] proposed a bimodality fusion approach for emotion recognition in the wild from video images. Their experimental results on the EmotiW2019 dataset gave a classification performance of around 63%. Lu et al. [21] proposed to utilize a spatiotemporal-feature fusion (MSFF) architecture for handling various feature characteristics for emotion recognition in the wild. Their proposed MSFF architecture was tested on the EmotionW2018 dataset and gave a classification performance of around 60%.

Chen et al. [22] offer another approach for emotion recognition research in the wild. This work describes the authors’ development of a large-scale and comprehensive dataset (termed HEU Emotion) for multimodal emotion recognition in the wild. Their HEU Emotion dataset contains videos from 19,004 video clips and 9951 subjects with ten emotions and multiple modalities (facial expressions, speech emotions and body postures). The authors performed some evaluations on the HEU dataset using conventional machine-learning and deep-learning approaches.

### 2.4. Other Research Works on In-The-Wild Scenarios

Other research works on in-the-wild scenarios include those of Hajavi &and Etemad [23] on speaker-recognition tasks and Nguyen et al. [24] for animal-recognition tasks. Hajavi and Etemad 2021 [23] proposed a Siamese network architecture using capsules and dynamic routing for speaker verification in the wild. Their experimental results on the VoxCeleb dataset gave an error rate (EER) of 3.14%. Nguyen et al. [24] proposed an approach utilizing deep learning and convolutional neural networks (CNNs) to monitor animals in their natural environments. Their experimental results on the Wildlife Spotter datasets gave a classification performance of around 90% in identifying three common animals in Australia (bird, rat and bandicoot).

## 3. Methodology

### Proposed Multimodal Sensor-Input Architecture with Deep Learning for AVSR in the Wild

**Figure 1:**
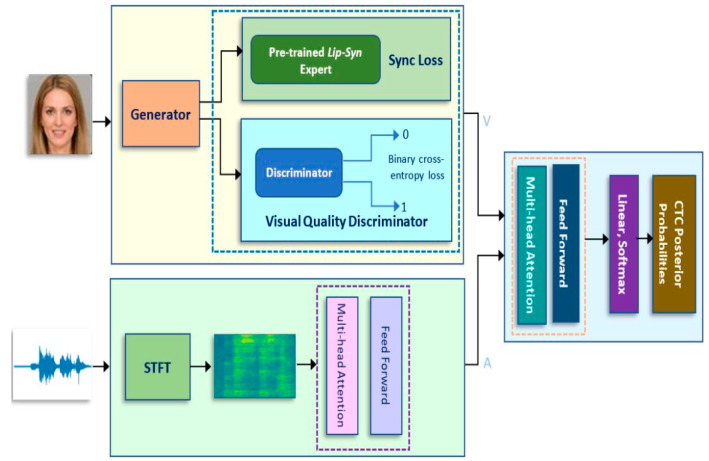
Proposed multimodal sensor-input architecture with deep learning for AVSR in the wild.

This section discusses the proposed multimodal sensor-input architecture with deep learning for AVSR in the wild. The first part of the section gives details on the multimodal sensor-input architecture and some of the implementation settings for each of the components in the different models. The latter part of the section gives details on the data-augmentation process to be incorporated into the AVSR architecture for improved performance. Some details on preprocessing are also discussed in this section. Figure 1 shows the proposed architecture for AVSR in the wild. The proposed architecture utilizes deep-learning approaches and self-attention modules based on the transformer model trained with CTC loss (TM–CTC; Afouras et al. [15]).

As shown in Figure 1, the model architecture for audiovisual speech recognition can be conceptually separated into three component models: (1) audio-only (AO); (2) video-only (VO); and (3) audiovisual. The top half (left side) of Figure 1 shows the video-only (VO) architecture, in which we used ResNet to process a sequence of visual images. We applied three-dimensional (3D) convolution and two-dimensional (2D) ResNet to the input image in order to match the audio frames. This allowed the sequence of input images to decrease in spatial size with depth and ensured that each input video frame was generated with a 512-dimension feature vector.

The bottom half (left side) of Figure 1 shows the audio-only (AO) architecture component. We obtained a spectrogram by using the short time Fourier transform (STFT) to process the audio signals for extracting audio features. The audio features took raw audio waves at 16 kHz as input and generated a vector representation every ten ms. The video clips had a frame rate of 25 fps and each video-input frame corresponded to four frames of the audio features.

The right half of Figure 1 shows the audio-visual (AV) architecture. We used the TM–CTC model for fusion of the audio and video data. The results were propagated through the self-attention and feedforward modules. The network generated the CTC posterior probability of each input frame and the entire stack architecture was trained to minimize the CTC losses. For preprocessing, in each video, we cropped a 112 × 112 patch to cover the area around the mouth. The cropped image patches were converted to grayscale and normalized based on the mean and variance of the training set. Similarly, the audio waveforms were normalized by subtracting their mean and dividing by the standard deviation.

**Figure 2:**
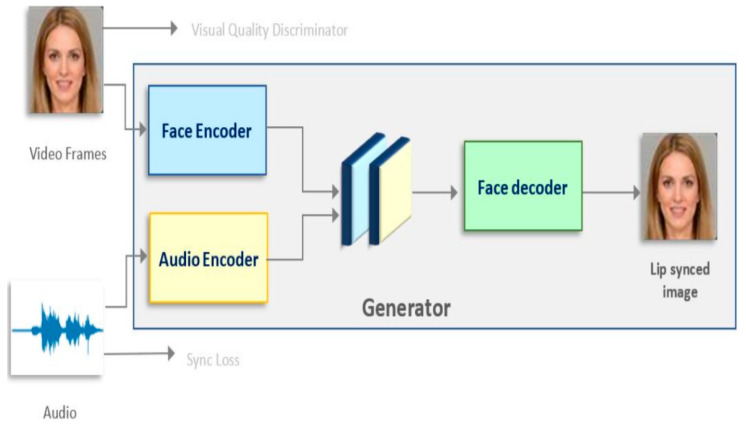
Proposed data-augmentation architecture using GANs for AVSR in the wild.

Figure 2 shows the proposed data-augmentation architecture using GANs for AVSR in the wild. These GANs form a neural network with both generative and adversarial aspects. The GAN network used for the experiments consisted of a generator and two discriminators. We processed the dataset through the GANs before it entered the TM–CTC architecture. In the GANs, the data first entered the generator, which consisted of two encoders and a decoder. The face encoder in the generator received video frames and generated intermediate features; the audio encoder in the generator received audio signals and generated audio intermediate features; the obtained face intermediate features and audio intermediate features were concatenated and sent to the face decoder for decoding. The final output was lip-shaped with audio-synchronized image frames (generated frames). The generated frames were fed into a discriminator, which determined whether the lip image and audio were synchronized and the quality of the lip video. After the GANs were processed, the new dataset was generated and then trained in the TM–CTC architecture.

In our experiments, we also used conventional approaches for the data augmentation. In the VO and AV architectures, we applied a horizontal flip to the image with a probability of 0.5 and removed random frames from the image sequence after the 112 × 112 random crop for data augmentation. In the audio-only and audio-visual architectures, we added clutter to each audio waveform with a 5-decibel signal-to-noise ratio and a probability of pn = 0.25 for the audio stream. The clutter was mixed from LRS2 with 20 different audio samples.

We used the Wave2Lip model proposed by Prajwal et al. [25] for the GAN data augmentation. The generator takes two parts of the model input, a sequence of video frames and an audio (melspectrogram segment) and fuses the features and generates frames that synchronize the lips with the audio. The architecture used two discriminators: (1) a discriminator to determine whether the generated lip image was synchronized with the audio; and (2) a discriminator to receive the lip image generated by the generator and the lip image synchronized with the audio to determine its authenticity and to produce better lip quality.

## 4. Experiment and Results

In this section, we first describe the details of the datasets and our experimental setup. This is followed by our experimental results, analysis and discussion.

### 4.1. Description of Datasets

This section gives a description of the datasets that were used in our experiments. We used the large-scale publicly available audio-visual dataset, Lip Reading Sentences 2 (LRS2) [26], as our main dataset for training and testing. The LRS2 dataset is known for its challenging scenarios, which feature substantial variations in head positions and lighting conditions. The LRS2 dataset contains over 224 h of data, consisting of 144,482 video clips extracted from BBC videos. The dataset is divided into training and validation/test sets. There are 96,318 video clips for pre-training, 45,839 video clips for training, 1082 video clips for validation and 1243 video clips for testing. The training data contain a vocabulary of over 40,000 words.

**Figure 3:**
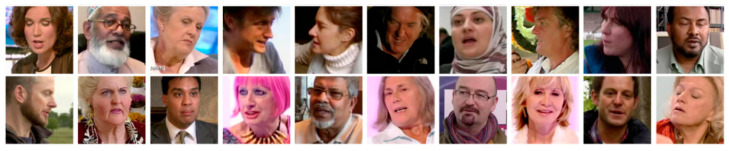
Example data from the LRS2 dataset [25].

**Figure 4:**
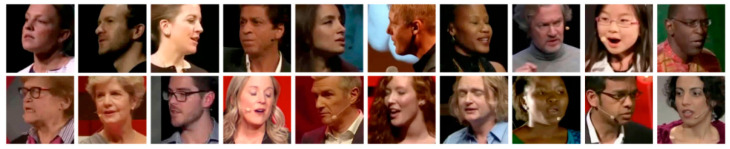
Example data from the LRS3 dataset [26].

In our experiments, we also used the LRS3–TED dataset. The LRS3 dataset [27] contains video clips collected from TED and TEDx talks. The LRS3 dataset contains over 438 h of data, consisting of 151,819 video clips extracted from TED and TEDx videos. There are 118,516 video clips for pre-training, 31,982 video clips for training/validation and 1321 video clips for testing. In our experiments, we also used datasets for training external language models. The language models were trained using a text corpus that contains the subtitles for the video clips. The corpus data contain a vocabulary of 26 million words. Figure 3 and Figure 4 show some example data from the LRS2 and LRS3 datasets, respectively.

### 4.2. Results and Discussion

In this sub-section, we first present and discuss the experimental results for the proposed architecture and the various settings of each of our components in the different models. Next, we show the results for the audio-only (AO), visual-only (VO and audio-visual (AV) settings. In addition, we analyze the relative contribution of each component (audio-only, visual-only and audio-visual settings) and compare and discuss the results of the different models.

For all experiments, we report the word-error rate (WER). In datasets LRS2 and LRS3, the smallest unit of the sentence is the word and the WER can measure the effectiveness of the system more accurately through words than other metrics. The WER can be calculated as

$$\mathrm{WER}=\frac{S+D+I}{N}$$

where *S*, *D*, *I* and *N* denote the number of substitutions, number of deletions, number of insertions and number of words in the reference, respectively. For the TM–CTC decoding, we used a beam search with a width of 100 (these values are determined on the LRS2 reserved-validation set) and a greedy search. We used character-level predictions with an output size of 40, including 26 alphabet characters and ten digits.

Our implementation was based on the Pytorch library and was trained on a NVIDIA A100 GPU with 80 GB of memory. The network models were trained using the ADAM optimizer. For all models, we used dropout and label smoothing.

Table 2 and Table 3 show the experimental results that were obtained for audio-only (AO), visual-only (VO) and audio-visual (AV) results of word-error rate (WER) tested on the LRS2 and LRS3 datasets, respectively. The experimental results are shown for both clean inputs and added noise. The tables also show the results for the proposed AVSR architectures and their components with and without the GAN data augmentation.

In the video-only (VO) modality, the best result for the WER was 55%, both with clean input and with added noise. This indicates that explicit linguistic consistency was not achieved in the VO modality. In the AO modality, trained with a clean audio input, the WER was able to achieve 8.7% when the external language model was added to the beam search. This indicates that the AO modality performed much better than the VO modality in the single-modality case. In the TM–CTC decoding, the beam search added an external language model and increased performance efficiency. We observed that the WER results were improved by up to 9.5%.

We used noisy audio for both the AO and the AV experiments. These noises were synthesized by adding babble noise to the raw audio. The recognition of speech in noisy environments is very challenging and this was reflected in the performance of our AO model, i.e., the WER was similar to the WER in the VO and the reduction in performance compared to the clean performance was over 49%. However, combining the two approaches caused significant improvements, with the WERs dropping by up to 33%. Furthermore, the AV model performed much better than the VO or AO models when the background sound was loud.

The performance improvements when the GAN component was used in the AVSR architecture can also be seen in the tables. When the speech signal contained substantial noise, the lip movements provided useful clues for speech recognition. For example, the WER was reduced from 52% to 27.9% for AO when using the audio-visual TM–CTC model. Furthermore, performance can be improved even when the audio signal does not contain noise (i.e., when it is clean). Compared to the AO model, similar results were obtained when using AV Wav2Lip+TM–CTC. The results show that the model can significantly improve the WER when using beam-search decoding with an external language model. Moreover, in noisy environments, the Wav2Lip+TM–CTC improves by 5.5% with the AO model. This indicates that the Wav2Lip+TM–CTC model has significant noise-rejection advantages over TM–CTC.

Without the audio input, the TM–CTC model is much better at lip reading for WERs. However, the Wav2Lip+TM–CTC model can process background noise better. Under large babble noise, the AO Wav2Lip+TM–CTC model performs significantly better than the TM–CTC model in both situations. This indicates that using Wav2Lip to synchronize the generated video character mouthing with the input speech can improve performance in audio language modeling. The results show that compared to greedy-search decoding, adding an external language model (+LM) to the beam search resulted in a better performance for both models.

For the large audio-visual datasets, LRS2 and LRS3, we found similarities in the results. The model-performance results that were good with LRS2 continued to be good and, sometimes, even better, with LRS3, despite their different data contents.

## 5. Conclusions

This paper investigated and contributed towards multimodal sensor-input AVSR architectures and research particularly for in-the-wild scenarios. This paper discussed the domains of ASR and AVSR research and proposed new improvements by incorporating data-augmentation techniques (conventional and GAN-based) into AVSR architectures in the wild. The experimental results using established large-scale LRS2 and LRS3 datasets validated the proposed architecture and showed that the proposed AVSR-architecture model and its various components, combined with the augmentation approach, enhanced the performance of the AVSR frameworks in the wild, making them particularly applicable to noisy datasets. Our future work will investigate this AVSR approach in combination with different GAN models for both audio and visual modalities.

## Figures and Tables

**Table 1:** Some representative works on multimodal-sensor architectures for ASR, AVSR and other applications in wild scenarios.

Category/Domain Area	Year	Main Contributions	Datasets	Reference
Research works on ASR in the wild	2022	Visual speech recognition in the wild—proposed zero-shot learning architecture for visual-only keyword spotting (KWS) tasks	LRS2 dataset	Stafylakis and Tzimiropoulos, 2018 [17]
	2021	Conversational-telephony speech recognition in the wild—evaluated three deep-learning architectures (time-delay neural network (TDNN), bidirectional long short-term memory (BLSTM), convolutional neural network BLSTM (CNN–BLSTM))	Switchboard and CallHome datasets	Han et al. [18]
	2018	In-the-wild ASR for Arabic language—submissions from 13 teams, best performance, with 80% accuracy, obtained using a combined approach of GANs and lexical information	Arabic MGB-3 Challenge dataset	Ali, Vogel and Renals [19]
Research works on AVSR in the wild	2020	In-the-wild AVSR for speech with overlapped speech (interfering speakers)—proposed time-delay neural networks (TDNNs) with lattice-free MMI (LF-MMI TDNN system), outperformed audio-only ASR baseline by 29% WER	LRS2 dataset	Yu et al. [14]
	2018	An AVSR deep-learning transformer models—proposed two transformer (TM) models and architectures for AVSR: (1) encoder–decoder-attention-structure TM architecture; and (2) self-attention TM stack architecture (TM–CTC)	LRS2-BBC dataset	Afouras et al. [15]
	2017	An AVSR watch, listen, attend and Spell (WLAS) network model with the ability to transcribe speech into characters	LRS and GRID datasets	Son Chung et al. [13]
Research works on emotion recognition in the wild	2019	Bimodal fusion approach for emotion recognition in the wild from video—experimental results from EmotiW2019 dataset gave a performance of 63%	EmotiW2019 dataset	Li et al. [20]
	2018	Spatiotemporal-feature fusion (MSFF) architecture—experimental results from EmotionW2018 dataset gave a performance of 60%	EmotionW2018 dataset	Lu et al. [21]
	2021	Dataset for multimodal emotion recognition in the wild (HEU Emotion)—videos from 19,004 video clips and 9,951 people with ten emotions and multiple modalities (facial expression, body posture and emo-tional speech)	HEU dataset	Chen et al. [22]
Research works on other in-the-wild applications	2021	Speaker recognition in the wild—Siamese network architecture using capsules and dynamic routing, experimental results gave an error rate (EER) of 3.14%	VoxCeleb dataset	Hajavi and Etemad [23]
	2017	Animal recognition in the wild—convolutional neural networks (CNNs) to monitor animals in their natural environments. Experimental results gave a classification performance of around 90% in identifying three common animals in Australia	Wildlife Spotter datasets	Nguyen et al. [24]

**Table 2:** Audio-only (AO), visual-only (VO) and audio-visual (AV) results of word-error rate (WER) tested on LRS2 dataset.

AVSR Architecture		Greedy Search	Beam Search (+LM)
		Clean Input	
TM–CTC+Wav2Lip GANs	AO	11.30%	8.70%
TM–CTC+Wav2Lip GANs	VO	73.00%	61.50%
TM–CTC+Wav2Lip GANs	AV	11.90%	8.40%
TM–CTC	AO	11.70%	8.90%
TM–CTC	VO	61.60%	55.00%
TM–CTC	AV	10.80%	7.10%
		Added Noise	
TM–CTC+Wav2Lip Gans	AO	60.20%	52%
TM–CTC+Wav2Lip Gans	AV	35.70%	27.90%
TM–CTC	AO	65.60%	56.10%
TM–CTC	AV	32.20%	23.70%

**Table 3:** Audio-only (AO), visual-only (VO) and audio-visual (AV) results of word-error rate (WER) tested on LRS3 dataset.

AVSR Architecture		Greedy Search	Beam Search (+LM)
		Clean Input	
TM–CTC+Wav2Lip GANs	AO	11.80%	9.80%
TM–CTC+Wav2Lip GANs	VO	88.00%	74.00%
TM–CTC+Wav2Lip GANs	AV	13.80%	12.60%
TM–CTC	AO	12.20%	10.50%
TM–CTC	VO	76.80%	67%
TM–CTC	AV	12.50%	11.30%
		Added Noise	
TM–CTC+Wav2Lip Gans	AO	56.00%	51.50%
TM–CTC+Wav2Lip Gans	AV	39.20%	31.90%
TM–CTC	AO	61.90%	55.40%
TM–CTC	AV	35.60%	27.70%

## Data Availability

Not applicable.
